# Effects of an essential amino acid mixture on behavioral and psychological symptoms of dementia and executive function in patients with Alzheimer's disease: A double‐blind, randomized, placebo‐controlled exploratory clinical trial

**DOI:** 10.1002/gps.5782

**Published:** 2022-08-05

**Authors:** Michihiro Takada, Shin Tanaka, Koh Tanaka, Tamao Tsukie, Masako Tsukamoto‐Yasui, Katsuya Suzuki, Yasushi Noguchi, Akira Imaizumi, Makoto Ishii, Takeshi Ikeuchi

**Affiliations:** ^1^ Ajinomoto Co., Inc. Tokyo Japan; ^2^ Mishima Hospital Niigata Japan; ^3^ Department of Molecular Genetics Brain Research Institute Niigata University Niigata Japan

**Keywords:** Alzheimer's disease, essential amino acids, executive function, Frontal Assessment Battery

## Abstract

**Background:**

The increasing number of dementia patients has become a global social problem. Amino acids are known to be used as precursors of neurotransmitters in the brain. Amino acid mixtures as a supplement may be used as a solution to Alzheimer's symptoms. This exploratory study evaluated the efficacy and safety of a mixture containing nine essential amino acids on behavioral and psychological symptoms of dementia (BPSD) and cognitive function in patients with Alzheimer's disease (AD).

**Design:**

We conducted a double‐blind, randomized, placebo‐controlled trial to evaluate the intervention effects of nine essential amino acid mixture for 28 days. A total of 36 patients with AD were enrolled in Japan. BPSD and cognitive function were evaluated by the Neuropsychiatric Inventory‐12 item (NPI‐12; the primary endpoint), Mini‐Mental State Examination (MMSE), Trail Making Test A (TMT‐A), Trail Making Test B (TMT‐B), Frontal Assessment Battery (FAB), and Clinical Dementia Rating Scale (CDR).

**Results:**

Compared with placebo, the amino acid mixture did not improve NPI‐12, MMSE, TMT‐A and B or CDR scores. However, the analysis of covariance revealed improved FAB scores in the amino acid mixture group as a secondary endpoint. There were four subjects with adverse events in each group.

**Conclusions:**

Our results did not show a beneficial effect of the mixture containing nine essential amino acids on BPSD as a primary endpoint; however, it may improve executive function in patients with AD.

## Key points

- A double‐blind randomized placebo‐controlled trial was conducted to evaluate the effect of the mixture containing nine essential amino acids in patients with Alzheimer's disease.
- Intervention with amino acids mixture did not improve the behavioral and psychological symptoms of dementia.
- It was suggested that executive function may be improved by intervention of amino acid mixture.

## 1 INTRODUCTION

The number of Alzheimer's disease (AD) patients is increasing worldwide, and there are concerns that it will have a very large impact on medical care, social problems from a public health perspective.^1^

It has been pointed out that behavioral and psychological symptoms of dementia (BPSD) has a large impact on the burden of long‐term care and leads to a diminished quality of life of caregivers.^2^ Therefore, it is important not only to treat the core cognitive symptoms but also to treat BPSD from the mild stages of AD and alleviate BPSD symptoms in these patients from the viewpoint of social implications, including long‐term care problems.

We focused on amino acids, which are constituents of protein, as candidates for a new intervention showing effectiveness in treating the symptoms of dementia, especially BPSD. Among the amino acids, essential amino acids pass through the blood‐brain barrier via amino acid transporters. These essential amino acids are used as precursors of neurotransmitters in the brain. Considering that imbalances in essential amino acids and branched‐chain amino acids (BCAAs) in the brain were observed in a longitudinal study with an AD mouse model,^3^ an intake of essential amino acids may affect AD symptoms.

Amino acid intake may also be associated with AD symptoms in humans because it has been reported that the amount of neurotransmitters is imbalanced in the brains of AD patients in clinical study.^4^, ^5^ It has been reported that protein intake is reduced in dementia patients^4^ and that asymptomatic nutritional deficiencies are caused by essential amino acid deficiencies in the pre‐mild cognitive impairment (MCI) stage.^6^ Longitudinal epidemiological studies of the population have reported that dietary amino acid intake is associated with future cognitive decline.^7^ In clinical studies examining blood levels, low blood BCAA levels have been reported to increase the risk of AD.^8^ Considering these previous studies, supplementation with essential amino acids, which are precursors of various neurotransmitters, may improve the imbalance of neurotransmitters in the brain and alleviate the symptoms of BPSD.

In this study, we conducted a clinical trial to provide an exploratory evaluation of the effects of intake of an essential amino acid mixture on cognitive function in patients with AD. We examined the temporary intervention effects of nine essential amino acids consisting of L‐leucine, L‐isoleucine, L‐valine, L‐threonine, L‐histidine, L‐lysine, L‐methionine, and L‐tryptophan since the effects of mixtures of these essential amino acids, with intakes of 3 or 6 g per day, on muscle synthesis and strength have been confirmed in previous studies, and the safety of their intake has also been confirmed.^9^, ^10^

## 2 METHODS

### 2.1 Ethical approval

Surrogates for all subjects provided informed consent for inclusion before the subjects participated in the study. When the physician judged that the patients could understand the contents of this study and were able to consent to participate based on his or her own free will, the patient's consent was also obtained. This study was conducted with the approval of the Human Subjects Study Review Committee of the Ethics Review Committee of Ajinomoto Co., Inc. (No. 2016‐031) and Niigata University (No. G2017‐0027).

### 2.2 Participants

Patients with AD who were going to the hospital were recruited. These patients were those who had been diagnosed with dementia and regularly visit the hospital, or those who have started going to our hospital and have a stable mental state. We enrolled patients who met all the inclusion criteria that included the following items: (1) patients who were Japanese individuals over 65 years old at the time of consent; (2) patients with a score of 15–26 on the Mini‐Mental State Examination (MMSE)^11^ and a diagnosis of AD based on the Diagnostic and Statistical Manual of Mental Disorders‐V; (3) patients with a score of 0.5–1 on the Clinical Dementia Rating Scale (Japanese version; CDR‐J)^12^ and judged by a physician to have mild AD; (4) patients who had one or more items on the Neuropsychiatric Inventory‐12 item (NPI‐12) that scored 4 or more points (severity*frequency)^13^, ^14^; and (5) patients who had been taking anti‐dementia medicine for more than 4 weeks or had not taken any anti‐dementia medicine.

We excluded patients if they met any of the following criteria: (1) had been administered or ingested medicines (or supplements) at least once a day that contained amino acids or ingredients that could affect cognitive function; (2) had less than 6 years of education in school; (3) had a history of alcoholism or were undergoing treatment for alcoholism; (4) were being treated for cancer or cirrhosis; (5) were getting dialysis; (6) were taking psychiatric medicines (however, if this dose was constant, registration was possible); and (7) had soy allergies.

Subject registration was carried out from 1 May 2017 to 28 December 2018.

### 2.3 Study design

We conducted a double‐blind, randomized, placebo‐controlled trial. This study was registered in the University Hospital Medical Information Network Clinical Trial Registry (UMIN000027186). The independent researcher in charge of allocation randomly assigned a placebo and amino acid mixture to boxes with a block size 4. The subjects were randomly assigned to the placebo and amino acid mixture groups stratified by the apathy score on the NPI‐12 (severity*frequency was ≥4 or <4) by taking the test food (indistinguishable, but either placebo or amino acid mixture) out of these boxes. This study was conducted at Mishima Hospital in Niigata, Japan. After deciding not to change the study design, an interim analysis was conducted to confirm safety because it was the first time that this amino acid mixture was used in patients with AD. No evaluation‐related biases were identified because the results of interim analysis were not shared with the researchers who evaluated the subjects and conducted the genetic analysis.

### 2.4 Interventions

The amino acid mixture, at 4.7 g/piece, was composed of nine essential amino acids. This amino acid mixture contained 1.61 g of leucine, 0.43 g of isoleucine, 0.44 g of valine, 0.37 g of threonine, 0.07 g of histidine hydrochloride, 0.67 g of lysine hydrochloride, 0.13 g of methionine, 0.03 g tryptophan, and 0.27 g of phenylalanine. As a placebo, the essential amino acids and sucrose fatty acid esters were replaced with powdered reduced malt starch syrup. The subjects ingested the test foods twice a day between meals with water for 28 days. During the intervention period, intake of amino acid/protein supplements, Docosahexaenoic acid, Eicosapentaenoic acid, and ginkgo biloba supplements was prohibited. Ingestion of test foods within 2 h after meals was prohibited. Eating within 1 h of ingesting the test food was also prohibited.

### 2.5 Outcomes and visit schedule

**FIGURE 1:**
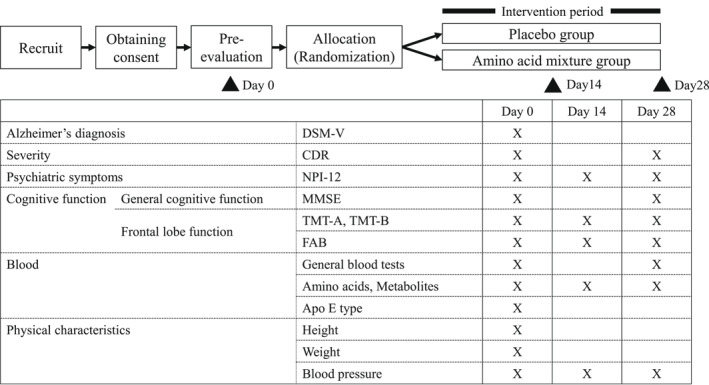
Trial schedule. Apo E, Apolipoprotein E; CDR, Clinical Dementia Rating Scale; DSM‐V, Diagnostic and Statistical Manual of Mental Disorders‐V; FAB, Frontal Assessment Battery; NPI‐12, Neuropsychiatric Inventory (NPI)‐12 item; TMT, Trail Making Test; X, evaluate outcomes

Figure 1 shows the measurement schedule for the outcomes. The genotype of *APOE* was measured, since it was reported that the genotype of *APOE* ε4 is associated with the symptoms of AD.^15^ The subjects ingested the test food from the day after the preingestion measurements (Day 0). Regarding the evaluation dates 14 and 28 days later, there was an allowable range from 3 days before to 3 days after the actual number of days since initiation of the study. The primary endpoint was the NPI‐12 total score,^13^, ^14^ which evaluates neuropsychiatric symptoms related to BPSD. The secondary endpoints were the subscores for each NPI‐12 item, MMSE scores^11^ to evaluate general cognitive function, Frontal Assessment Battery (FAB),^16^ Trail Making Test A & B (TMT‐A and B) scores^17^, ^18^, ^19^ to assess frontal lobe function, and CDR‐J scores^12^ to assess dementia severity. As safety evaluation outcomes, the evaluation of overall safety by physicians and the number of patients experiencing adverse events were recorded. The plasma concentrations of the amino acids and other metabolites were also evaluated.

### 2.6 Measurements

During the study period, the caregivers (surrogates) involved in the evaluation of the NPI‐12 (Each subscore was calculated with a maximum of 12 points of severity*frequency, and the total score of NPI‐12 was calculated with a maximum of 144 points) and the evaluators involved in obtaining MMSE, FAB, TMT‐A, TMT‐B and CDR scores were not allowed to change.

From 10 h before a blood draw, the subjects were prohibited from ingesting amino acid beverages and food. On Day 0 (before the initiation of the ingestion of test food) and Day 28, 15 ml of blood was drawn from each subject. In consideration of amino acid stability, blood for the amino acid analyses was immediately mixed by inversion, and the blood collection tubes were immediately cooled, and cryopreserved at −75°C after centrifugation and dispensing. The concentrations of amino acids and metabolites in the sample and laboratory test values were measured. *APOE* genotypes were analyzed at Niigata University.

### 2.7 Statistical analysis

With reference to the report by Kawanabe et al.^20^ the standard deviation (SD) of the NPI‐12 total score was assumed to be 10.0. We determined that a sample of 34 subjects would provide this study with 80% statistical power to detect a 10‐point mean difference between groups in the NPI‐12 total score with a two‐sided significance level of 5%.

All assigned subjects with at least one postintervention effective data were defined as the full analysis set (FAS).

The primary endpoint, NPI‐12 score, was compared between groups using an unpaired *t* test for the change values from Day 0, and analysis of covariance (ANCOVA) adjusted for baseline NPI‐12 scores (preingestion), years of education, and presence/absence of ApoE4 (2*2, 2*3, 3*3 or 3*4, 4*4), as these have been reported to affect cognitive function.^21^ ANCOVA was performed as a post hoc analysis. Regarding the secondary endpoints, MMSE, TMT‐A, TMT‐B, and FAB scores were also analyzed in the same manner as NPI‐12 scores. For CDR, the Wilcoxon rank‐sum test was used to compare groups. For plasma amino acid concentrations and other metabolite concentrations, unpaired *t* tests were performed to compare between groups. The laboratory test values were compared between groups 28 days later.

The above analysis plan and standard for handling patient data were fixed before the key opening for the interim analysis. The results are reported as the mean ± SD. In the analysis of the effects, the differences between groups in the changes from Day 0 and their 95% confidence intervals (95% CIs) and *p* values are reported. Based on ANCOVA results, the estimated effects, 95% CIs, and *p* values are reported. All analyses were conducted at a two‐sided 5% significance level without adjusting for multiple comparisons because of the exploratory nature of the study. All statistical analyses were performed by R ver.3.4.3 (R Foundation for Statistical Computing).

## 3 RESULTS

### 3.1 Subjects

**FIGURE 2:**
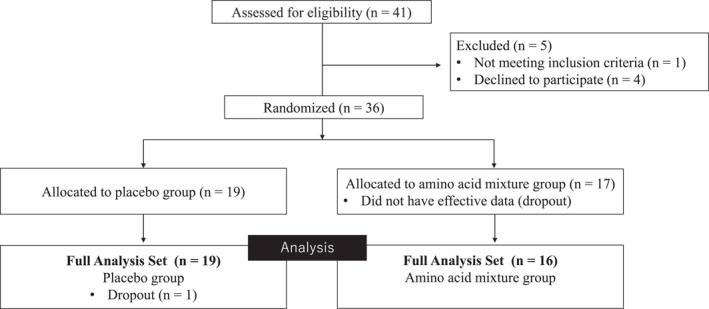
Flow diagram of subjects

Figure 2 shows the flowchart of the subjects after enrollment in this study. Among 41 patients considered for inclusion, 36 were enrolled and assigned to one of the two groups. After the intervention, one patient who developed pneumonia and sepsis was discontinued. However, because this patient had no postintervention data, the adverse events were included in the aggregate, and the remaining data were excluded from the FAS. After the interventions, there was one dropout in the placebo group.

### 3.2 Background characteristics

Regarding the status of intake compliance, 6 of the 19 subjects in the placebo group missed their scheduled intake. The number of times these six people forgot to ingest their food was 2.5 ± 1.6 times. Five of the 18 subjects in the amino acid mixture group missed their scheduled intake. The number of times these five people forgot to ingest their food was 2.0 ± 1.2 times.

The background characteristics of the subjects is shown in Table 1. There was a significant difference between the groups in the years of education. The 4*4 type of *APOE*, which has been suggested to be associated with the onset of AD, was biased toward the amino acid mixture group, with 0 in the placebo group and 2 in the amino acid mixture group.

**TABLE 1:** Baseline characteristics of subjects (Day 0)

Mean ± standard deviation	Placebo group	Amino acid mixture group
Number, percentage within the group	*N* = 19	*N* = 16
Age (year)	80.2 ± 5.4	82.0 ± 5.8
Sex, male	4, 21%	3, 19%
Years of education (year)	10.6 ± 1.9	9.2 ± 1.5
Disease duration (year)^a^	2.5 ± 1.4	4.0 ± 3.5
Height (cm)	149.9 ± 7.6	147.1 ± 7.0
Weight (kg)	47.9 ± 8.4	48.7 ± 6.5
Body mass index (kg/m^2^)	21.2 ± 2.8	22.5 ± 2.6
Systolic blood pressure (mmHg)	137.7 ± 17.7	139.2 ± 19.1
Diastolic blood pressure (mmHg)	68.0 ± 13.2	73.8 ± 9.2
Taking anti‐dementia medicine		
Donepezil	6, 32%	8, 50%
Galantamine	2, 11%	0, 0%
Rivastigmine	0, 0%	1, 6%
Memantine	0, 0%	3, 19%
No medicine	11, 58%	6, 38%
Taking sleeping medicine	5, 26%	3, 19%
Taking psychotropic medicine	2, 11%	2, 13%
NPI‐12 score	9.7 ± 6.8	12.1 ± 10.0
MMSE score	19.7 ± 3.3	20.1 ± 3.1
TMT‐A (s)	96.4 ± 39.2	100.6 ± 27.4
TMT‐B (s)	252.6 ± 84.3	228.9 ± 80.0
FAB score	10.9 ± 2.4	10.9 ± 3.1
CDR score		
0.5	7, 37%	7, 44%
1	12, 63%	9, 56%
*APOE* genotype		
2*2	0, 0%	1, 6%
2*3	2, 11%	1, 6%
3*3	10, 53%	4, 25%
3*4 (with ApoE4)	7, 37%	8, 50%
4*4 (with ApoE4)	0, 0%	2, 13%
Blood albumin concentration (g/dl)	4.1 ± 0.5	4.3 ± 0.4

Abbreviations: CDR, Clinical Dementia Rating Scale; FAB, Frontal Assessment Battery; MMSE, Mini‐Mental State Examination; NPI‐12, Neuropsychiatric Inventory‐12 item; TMT‐A, Trail Making Test A; TMT‐B, Trail Making Test B.

^a^ Disease duration is shown in years, assuming 365 days per year.

### 3.3 Effectiveness for cognitive function

The results for both the primary (NPI‐12 scores) and secondary endpoints are shown in Table 2. The NPI‐12 scores before the intervention were 9.7 ± 6.8 in the placebo group and 12.2 ± 10.0 in the amino acid mixture group, showing no significant difference between the groups. There was no significant difference between the groups on Day 14 and Day 28. No significant difference was confirmed in the comparison between groups for each subscore of NPI‐12 (Table S1).

**TABLE 2:** Effects on BPSD and cognitive functions

			Placebo group		Amino acid mixture group	
			*N*	Mean ± SD	*N*	Mean ± SD
NPI‐12	Day 0	(Score)	19	9.7 ± 6.8	16	12.1 ± 10.0
	Day 14	(Score)	17	7.2 ± 6.9	13	9.3 ± 6.5
		Change from Day 0	17	−1.9 ± 2.6	13	−1.8 ± 2.6
		Group difference [95% CI], *t* test *p* value	0.0 [−1.9, 2.0], *p* = 0.970			
		ANCOVA group difference estimate [95% CI], *p* value	−0.2 [−2.4, 1.9], *p* = 0.833			
	Day 28	(Score)	17	7.3 ± 7.9	15	10.7 ± 11.2
		Change from Day 0	17	−3.0 ± 3.7	15	−1.7 ± 2.8
		Group difference [95% CI], *t* test *p* value	1.3 [−1.1, 3.7], *p* = 0.268			
		ANCOVA group difference estimate [95% CI], *p* value	1.2 [−1.7, 4.1], *p* = 0.410			
MMSE	Day 0	(Score)	19	19.7 ± 3.3	16	20.1 ± 3.1
	Day 28	(Score)	18	20.4 ± 3.7	16	20.0 ± 3.4
		Change from Day 0	18	0.7 ± 2.1	16	−0.1 ± 2.9
		Group difference [95% CI], *t* test *p* value	−0.8 [−2.6, 0.9], *p* = 0.333			
		ANCOVA group difference estimate [95% CI], *p* value	0.8 [−1.2, 2.7], *p* = 0.422			
TMT‐A	Day 0	(Score)	19	96.4 ± 39.2	16	100.6 ± 27.4
	Day 14	(Score)	19	94.6 ± 43.3	15	95.7 ± 34.7
		Change from Day 0	19	−1.7 ± 27.0	15	−7.6 ± 23.6
		Group difference [95% CI], *t* test *p* value	−5.9 [−23.8, 12.1], *p* = 0.511			
		ANCOVA group difference estimate [95% CI], *p* value	−18.0 [−36.9, 1.0], *p* = 0.062			
	Day 28	(Score)	18	84.3 ± 34.9	16	95.6 ± 39.8
		Change from Day 0	18	−9.2 ± 21.7	16	−5.1 ± 27.7
		Group difference [95% CI], *t* test *p* value	4.1 [−13.2, 21.4], *p* = 0.632			
		ANCOVA group difference estimate [95% CI], *p* value	0.1 [−20.1, 20.2], *p* = 0.995			
TMT‐B	Day 0	(Score)	18	252.6 ± 84.3	16	228.9 ± 80.0
	Day 14	(Score)	19	242.6 ± 89.2	15	246.3 ± 75.5
		Change from Day 0	18	−0.3 ± 59.5	15	14.1 ± 82.8
		Group difference [95% CI], *t* test *p* value	14.4 [−36.2, 65.0], *p* = 0.566			
		ANCOVA group difference estimate [95% CI], *p* value	6.5 [−48.8, 61.7], *p* = 0.813			
	Day 28	(Score)	18	232.5 ± 80.3	16	213.8 ± 73.6
		Change from Day 0	17	−21.3 ± 70.0	16	−15.1 ± 82.0
		Group difference [95% CI], *t* test *p* value	6.2 [−47.8, 60.2], *p* = 0.815			
		ANCOVA group difference estimate [95% CI], *p* value	−9.5 [−67.5, 48.5], *p* = 0.740			
FAB	Day 0	(Score)	19	10.9 ± 2.4	16	10.9 ± 3.1
	Day 14	(Score)	19	11.4 ± 2.5	15	11.7 ± 3.2
		Change from Day 0	19	0.4 ± 2.0	15	0.7 ± 1.7
		Group difference [95% CI], *t* test *p* value	0.2 [−1.1, 1.6], *p* = 0.709			
		ANCOVA group difference estimate [95% CI], *p* value	1.2 [−0.1, 2.5], *p* = 0.073			
	Day 28	(Score)	18	11.7 ± 2.8	15	12.5 ± 3.0
		Change from Day 0	18	0.7 ± 1.5	15	1.1 ± 1.8
		Group difference [95% CI], *t* test *p* value	0.5 [−0.7, 1.7], *p* = 0.428			
		ANCOVA group difference estimate [95% CI], *p* value	1.5 [0.3, 2.8], *p* = 0.014			
CDR	Day 0	Score 0	0, 0%		0, 0%	
		Score 0.5	7, 37%		7, 44%	
		Score 1	12, 63%		9, 56%	
	Day 28	Score 0	1, 6%		0, 0%	
		Score 0.5	7, 41%		7, 44%	
		Score 1	9, 53%		9, 56%	
		Wilcoxon rank‐sum test *p* value	*p* = 0.757			

*Note*: Group difference: differences between groups (amino acid mixture group—placebo group) in the change from Day 0 and their 95% confidence intervals (95% CI). *t* test: comparison between groups in the change from Day 0 by unpaired *t* test. ANCOVA: analysis of covariance adjusted for baseline value (Day 0), years of education and ApoE4 type. Percentages show the proportions within the group.

Abbreviations: BPSD, behavioral and psychological symptoms of dementia; CDR, Clinical Dementia Rating Scale; FAB, Frontal Assessment Battery; MMSE, Mini‐Mental State Examination; NPI‐12, Neuropsychiatric Inventory‐12 item; SD, standard deviation; TMT‐A, Trail Making Test A; TMT‐B, Trail Making Test B.

There were no significant differences in MMSE, TMT‐A, TMT‐B and CDR scores between the groups at the time of evaluation after the intervention.

Although there was no significant difference in FAB scores based on unpaired *t* tests on Day 14 and Day 28, ANCOVA showed that there may be differences (estimate: 1.5 [95% CI: 0.3–2.8], *p* = 0.014) between groups on Day 28. The results for the FAB subscore are shown in Table S2. Among the subsets of the FAB, the amount of change in subset‐(3) score in the amino acid mixture group on Day 14 was larger (0.5 [95% CI: 0.0, 1.0]) than that in the placebo group. ANCOVA showed that there may be differences (estimate: 0.6 [95% CI: 0.1, 1.1]) in subset‐(3) of the FAB between groups on Day 28.

### 3.4 Plasma amino acid concentrations

Threonine concentrations in plasma were significantly higher in the amino acid mixture group than in the placebo group on Day 14 (*p* < 0.001) and Day 28 (*p* = 0.009), and alanine was also significantly higher in the amino acid mixture group on Day 14 (*p* = 0.042).

### 3.5 Safety evaluations

Regarding the overall safety level, there were no safety problems in either the placebo or the amino acid mixture group on Day 14 and Day 28. Adverse events were observed in four subjects in the placebo group and four subjects in the amino acid mixture group. Regarding serious adverse events with hospitalization, one patient was hospitalized for pneumonia in the placebo group, and one patient was hospitalized for pneumonia/sepsis in the amino acid mixture group. None of the adverse events were judged to have a causal relationship with the interventions by the physician. No laboratory test values suggesting concern for safety were observed.

## 4 DISCUSSION

In this study, we conducted an exploratory evaluation of the intervention effects of the intake of nine amino acids in patients with AD. There was no significant difference between the groups in NPI‐12 scores, which were used to evaluate the neuropsychiatric symptoms related to BPSD and set as the primary endpoint.

On the other hand, with the FAB, which was an evaluation index of executive function set as one of the secondary evaluation items, the results suggested that the frontal lobe‐related symptoms were alleviated^16^ by intake of the nine amino acids. Considering that the amino acid mixture intervention showed efficacy on the FAB only and subset‐(3) of the FAB, it could be suggested that the amino acid mixture had a moderating effect on the decline in executive function. Executive function has been associated with working memory, attention, concentration and cognitive flexibility.^22^ It is considered that there are no safety concerns.

The results of the analysis of plasma amino acid levels suggested that the deficient threonine was ameliorated by the amino acid mixture intervention and that the BCAAs contained in the amino acid mixture were directly converted to alanine. However, to assess the effects of this intervention on plasma amino acid levels, additional studies are needed since blood was drawn under fasting conditions for appropriately evaluating clinical laboratory test values.

The amino acid mixture was high in leucine. In a previous study with mice, leucine was reported to suppress the influx of kynurenine, which is involved in brain inflammation, by acting on the amino acid transporter LAT1 expressed in the blood–brain barrier.^23^ In this previous study, the antidepressant effects of leucine on inflammation‐induced depression were investigated. Since there is a hypothesis that dementia is also related to brain inflammation, it is possible that leucine was effective for cognitive function by a similar mechanism. In addition, in clinical trials evaluating the nutritional intervention effects of a combination of medium‐chain fatty acids, leucine, and vitamin D3 in humans, effects on improving cognitive function in frail older people has also been confirmed.^24^

In addition, this amino acid mixture is characterized by containing a large amount of lysine at 14.3%. In previous studies, lysine deficiency suppressed growth hormone secretion.^25^ Since it has been reported that a decrease in growth hormone was associated with a decrease in cognitive function,^26^ this amino acid mixture may be involved in efficacy by assisting in preventing lysine deficiency. In fact, it has also been shown that lysine levels in the brain are reduced in AD patients.^27^ Since lysine crosses the blood‐brain barrier^28^ and dietary intake is known to be positively correlated with brain concentration,^29^ it is possible that lysine intake contributes to an increase in lysine concentrations in the brain.

There are limits to this research. First, due to the exploratory research, it had the disadvantages of a small sample size and a short intervention period. Second, it is possible that efficacy could not be detected in the primary endpoint since the population had a low NPI score. Third, the distress score was not measured in this study. Fourth, because this was an exploratory study, no adjustments for multiple comparisons were conducted in the statistical analyses between outcomes.

In conclusion, based on the primary endpoint, NPI‐12 scores, the intervention was not effective. However, based on the secondary endpoint, FAB scores associated with executive function, there was a symptom‐relieving effect by the amino acid mixture intervention. Since this was an exploratory study, verification of this research will be important in the future.

## CONFLICT OF INTEREST

Michihiro Takada, Masako Tsukamoto‐Yasui, Katsuya Suzuki, Akira Imaizumi, Yasushi Noguchi, and Makoto Ishii are employees of Ajinomoto Co., Inc. The test foods were provided by Ajinomoto Co., Inc. Shin Tanaka and Takeshi Ikeuchi received grants from Ajinomoto Co., Inc.

## Supporting information

Supporting Information S1

Supporting Information S2

## Data Availability

The data that support the findings of this study are available on request from the corresponding author. The data are not publicly available due to privacy or ethical restrictions. However, there may be restrictions on the provision of data because the subject has not consented to the use of the data for other purposes.
