# Supplementary material for: Effects of an essential amino acid mixture on behavioral and psychological symptoms of dementia and executive function in patients with Alzheimer's disease: A double‐blind, randomized, placebo‐controlled exploratory clinical trial
Source: Int J Geriatr Psychiatry. 2022 Aug 5;37(9):10.1002/gps.5782. doi: 10.1002/gps.5782 (PMC9544995; doi:10.1002/gps.5782)
Supplement: Supplementary file 1 — Supporting Information S1 [file GPS-37-0-s002.docx]

Supplemental Table 1. NPI-12 sub-items.

| Score (severity*frequency) | |  | Placebo group | | | |  | Active group | | | |  |  |
| --- | --- | --- | --- | --- | --- | --- | --- | --- | --- | --- | --- | --- | --- |
|  |  |  | N | Mean | ± | SD |  | N | Mean | ± | SD |  | p value |
| Delusions | Day 0 |  | 19 | 0.2 | ± | 0.7 |  | 16 | 0.4 | ± | 1.5 |  |  |
|  | Day 14 |  | 19 | 0.0 | ± | 0.0 |  | 13 | 0.0 | ± | 0.0 |  | --- |
|  | Day 28 |  | 17 | 0.0 | ± | 0.0 |  | 16 | 0.4 | ± | 1.5 |  | 0.310 |
|  | change values (Day14-Day0) |  | 19 | -0.2 | ± | 0.7 |  | 13 | 0.0 | ± | 0.0 |  | 0.417 |
|  | change values (Day28-Day0) |  | 17 | -0.2 | ± | 0.7 |  | 16 | 0.0 | ± | 0.0 |  | 0.340 |
| Hallucinations | Day 0 |  | 19 | 0.0 | ± | 0.0 |  | 16 | 0.0 | ± | 0.0 |  |  |
|  | Day 14 |  | 19 | 0.0 | ± | 0.0 |  | 13 | 0.0 | ± | 0.0 |  | --- |
|  | Day 28 |  | 17 | 0.1 | ± | 0.2 |  | 16 | 0.0 | ± | 0.0 |  | 0.340 |
|  | change values (Day14-Day0) |  | 19 | 0.0 | ± | 0.0 |  | 13 | 0.0 | ± | 0.0 |  | --- |
|  | change values (Day28-Day0) |  | 17 | 0.1 | ± | 0.2 |  | 16 | 0.0 | ± | 0.0 |  | 0.340 |
| Agitation/Aggression | Day 0 |  | 19 | 0.5 | ± | 1.3 |  | 16 | 1.4 | ± | 3.0 |  |  |
|  | Day 14 |  | 19 | 0.2 | ± | 0.9 |  | 13 | 0.5 | ± | 1.2 |  | 0.507 |
|  | Day 28 |  | 17 | 0.2 | ± | 0.7 |  | 16 | 1.0 | ± | 3.0 |  | 0.285 |
|  | change values (Day14-Day0) |  | 19 | -0.3 | ± | 1.6 |  | 13 | -0.4 | ± | 1.2 |  | 0.820 |
|  | change values (Day28-Day0) |  | 17 | -0.1 | ± | 1.3 |  | 16 | -0.4 | ± | 1.0 |  | 0.423 |
| Depression/Dysphoria | Day 0 |  | 19 | 0.7 | ± | 2.2 |  | 16 | 0.3 | ± | 1.0 |  |  |
|  | Day 14 |  | 19 | 0.8 | ± | 2.5 |  | 13 | 0.0 | ± | 0.0 |  | 0.241 |
|  | Day 28 |  | 17 | 0.6 | ± | 2.0 |  | 16 | 0.3 | ± | 1.0 |  | 0.620 |
|  | change values (Day14-Day0) |  | 19 | 0.1 | ± | 0.5 |  | 13 | -0.3 | ± | 1.1 |  | 0.155 |
|  | change values (Day28-Day0) |  | 17 | -0.2 | ± | 1.0 |  | 16 | 0.1 | ± | 0.3 |  | 0.243 |
| Anxiety | Day 0 |  | 19 | 0.8 | ± | 2.0 |  | 16 | 1.6 | ± | 2.2 |  |  |
|  | Day 14 |  | 19 | 0.7 | ± | 2.0 |  | 13 | 1.5 | ± | 1.9 |  | 0.316 |
|  | Day 28 |  | 17 | 0.6 | ± | 1.9 |  | 16 | 1.5 | ± | 3.1 |  | 0.318 |
|  | change values (Day14-Day0) |  | 19 | -0.1 | ± | 1.3 |  | 13 | 0.1 | ± | 1.0 |  | 0.674 |
|  | change values (Day28-Day0) |  | 17 | -0.4 | ± | 0.9 |  | 16 | -0.1 | ± | 1.6 |  | 0.609 |
| Elation/Euphoria | Day 0 |  | 19 | 0.0 | ± | 0.0 |  | 16 | 0.0 | ± | 0.0 |  |  |
|  | Day 14 |  | 19 | 0.0 | ± | 0.0 |  | 13 | 0.0 | ± | 0.0 |  | --- |
|  | Day 28 |  | 17 | 0.0 | ± | 0.0 |  | 16 | 0.0 | ± | 0.0 |  | --- |
|  | change values (Day14-Day0) |  | 19 | 0.0 | ± | 0.0 |  | 13 | 0.0 | ± | 0.0 |  | --- |
|  | change values (Day28-Day0) |  | 17 | 0.0 | ± | 0.0 |  | 16 | 0.0 | ± | 0.0 |  | --- |
| Apathy/Indifference | Day 0 |  | 19 | 5.0 | ± | 2.1 |  | 16 | 4.9 | ± | 2.2 |  |  |
|  | Day 14 |  | 19 | 4.9 | ± | 2.3 |  | 13 | 4.6 | ± | 2.3 |  | 0.694 |
|  | Day 28 |  | 17 | 4.3 | ± | 2.0 |  | 16 | 4.7 | ± | 2.3 |  | 0.605 |
|  | change values (Day14-Day0) |  | 19 | -0.1 | ± | 2.0 |  | 13 | -0.2 | ± | 0.4 |  | 0.858 |
|  | change values (Day28-Day0) |  | 17 | -0.9 | ± | 1.8 |  | 16 | -0.2 | ± | 0.4 |  | 0.116 |
| Disinhibition | Day 0 |  | 19 | 0.0 | ± | 0.0 |  | 16 | 0.0 | ± | 0.0 |  |  |
|  | Day 14 |  | 19 | 0.0 | ± | 0.0 |  | 13 | 0.0 | ± | 0.0 |  |  |
|  | Day 28 |  | 17 | 0.0 | ± | 0.0 |  | 16 | 0.0 | ± | 0.0 |  | --- |
|  | change values (Day14-Day0) |  | 19 | 0.0 | ± | 0.0 |  | 13 | 0.0 | ± | 0.0 |  | --- |
|  | change values (Day28-Day0) |  | 17 | 0.0 | ± | 0.0 |  | 16 | 0.0 | ± | 0.0 |  | --- |
| Irritability/Lability | Day 0 |  | 19 | 0.1 | ± | 0.2 |  | 16 | 0.0 | ± | 0.0 |  |  |
|  | Day 14 |  | 19 | 0.0 | ± | 0.0 |  | 13 | 0.0 | ± | 0.0 |  | --- |
|  | Day 28 |  | 17 | 0.0 | ± | 0.0 |  | 16 | 0.0 | ± | 0.0 |  | --- |
|  | change values (Day14-Day0) |  | 19 | -0.1 | ± | 0.2 |  | 13 | 0.0 | ± | 0.0 |  | 0.417 |
|  | change values (Day28-Day0) |  | 17 | -0.1 | ± | 0.2 |  | 16 | 0.0 | ± | 0.0 |  | 0.340 |
| Aberrant Motor Behavior | Day 0 |  | 18 | 0.0 | ± | 0.0 |  | 16 | 0.5 | ± | 2.0 |  |  |
|  | Day 14 |  | 19 | 0.0 | ± | 0.0 |  | 13 | 0.6 | ± | 2.2 |  | 0.233 |
|  | Day 28 |  | 17 | 0.0 | ± | 0.0 |  | 16 | 0.5 | ± | 2.0 |  | 0.310 |
|  | change values (Day14-Day0) |  | 18 | 0.0 | ± | 0.0 |  | 13 | 0.0 | ± | 0.0 |  | --- |
|  | change values (Day28-Day0) |  | 16 | 0.0 | ± | 0.0 |  | 16 | 0.0 | ± | 0.0 |  | --- |
| Sleep | Day 0 |  | 19 | 0.8 | ± | 2.1 |  | 16 | 1.8 | ± | 2.8 |  |  |
|  | Day 14 |  | 19 | 0.4 | ± | 1.4 |  | 13 | 1.4 | ± | 2.5 |  | 0.146 |
|  | Day 28 |  | 17 | 0.4 | ± | 1.2 |  | 16 | 1.2 | ± | 2.3 |  | 0.234 |
|  | change values (Day14-Day0) |  | 19 | -0.5 | ± | 1.2 |  | 13 | -0.5 | ± | 1.4 |  | 0.979 |
|  | change values (Day28-Day0) |  | 17 | -0.5 | ± | 1.5 |  | 16 | -0.6 | ± | 1.6 |  | 0.953 |
| Appetite and  eating disorders | Day 0 |  | 19 | 1.9 | ± | 2.9 |  | 16 | 1.3 | ± | 2.5 |  |  |
|  | Day 14 |  | 19 | 0.8 | ± | 2.1 |  | 13 | 0.8 | ± | 1.9 |  | 0.920 |
|  | Day 28 |  | 17 | 1.2 | ± | 2.7 |  | 16 | 0.8 | ± | 1.8 |  | 0.595 |
|  | change values (Day14-Day0) |  | 19 | -1.1 | ± | 2.7 |  | 13 | -0.6 | ± | 2.2 |  | 0.593 |
|  | change values (Day28-Day0) |  | 17 | -1.0 | ± | 2.4 |  | 16 | -0.6 | ± | 2.0 |  | 0.572 |

*t*-test: Comparison between groups by unpaired *t*-test. NPI-12: Neuropsychiatric Inventory-12 item, SD: Standard Deviation. As for the result displayed as ---, there was no variation in the data and the statistical tests could not be performed.
