# Supplementary material for: Effects of an essential amino acid mixture on behavioral and psychological symptoms of dementia and executive function in patients with Alzheimer's disease: A double‐blind, randomized, placebo‐controlled exploratory clinical trial
Source: Int J Geriatr Psychiatry. 2022 Aug 5;37(9):10.1002/gps.5782. doi: 10.1002/gps.5782 (PMC9544995; doi:10.1002/gps.5782)
Supplement: Supplementary file 2 — Supporting Information S2 [file GPS-37-0-s001.docx]

Supplemental Table 2. FAB subset results

|  |  |  |  |  | Placebo group | | | |  | Amino acid mixture group | | | |
| --- | --- | --- | --- | --- | --- | --- | --- | --- | --- | --- | --- | --- | --- |
|  |  |  |  |  | N | Mean | ± | SD |  | N | Mean | ± | SD |
| (1)  Similarities |  | Day 0 | (Score) |  | 19 | 1.3 | ± | 0.9 |  | 16 | 1.1 | ± | 1.0 |
|  |  | Day 14 | (Score) |  | 19 | 1.5 | ± | 1.1 |  | 15 | 1.5 | ± | 1.2 |
|  |  |  | Change from Day 0 |  | 19 | 0.3 | ± | 0.9 |  | 15 | 0.3 | ± | 1.0 |
|  |  |  | Group difference [95% CI], *t* test p value |  | 0.0 [-0.6, 0.6], p = 0.991 | | | | | | | | |
|  |  |  | ANCOVA group difference estimate [95% CI], p value |  | 0.4 [-0.3, 1.1], p = 0.307 | | | | | | | | |
|  |  | Day 28 | (Score) |  | 18 | 1.4 | ± | 1.0 |  | 16 | 1.3 | ± | 1.1 |
|  |  |  | Change from Day 0 |  | 18 | 0.2 | ± | 0.9 |  | 16 | 0.2 | ± | 0.8 |
|  |  |  | Group difference [95% CI], *t* test p value |  | 0.0 [-0.6, 0.6], p = 0.907 | | | | | | | | |
|  |  |  | ANCOVA group difference estimate [95% CI], p value |  | 0.4 [-0.3, 1.0], p = 0.233 | | | | | | | | |
| (2)  Verbal fluency |  | Day 0 | (Score) |  | 19 | 1.6 | ± | 0.8 |  | 16 | 1.6 | ± | 1.0 |
|  |  | Day 14 | (Score) |  | 19 | 1.2 | ± | 0.9 |  | 15 | 1.5 | ± | 0.9 |
|  |  |  | Change from Day 0 |  | 19 | -0.4 | ± | 0.6 |  | 15 | -0.2 | ± | 0.9 |
|  |  |  | Group difference [95% CI], *t* test p value |  | 0.2 [-0.3, 0.8], p = 0.413 | | | | | | | | |
|  |  |  | ANCOVA group difference estimate [95% CI], p value |  | 0.5 [-0.1, 1.1], p = 0.094 | | | | | | | | |
|  |  | Day 28 | (Score) |  | 18 | 1.8 | ± | 0.7 |  | 16 | 1.4 | ± | 0.8 |
|  |  |  | Change from Day 0 |  | 18 | 0.1 | ± | 0.6 |  | 16 | -0.2 | ± | 0.8 |
|  |  |  | Group difference [95% CI], *t* test p value |  | -0.2 [-0.8, 0.3], p = 0.344 | | | | | | | | |
|  |  |  | ANCOVA group difference estimate [95% CI], p value |  | 0.0 [-0.4, 0.5], p = 0.881 | | | | | | | | |
| (3)  Luria motor sequences |  | Day 0 | (Score) |  | 19 | 1.3 | ± | 0.6 |  | 16 | 1.3 | ± | 0.7 |
|  |  | Day 14 | (Score) |  | 19 | 1.3 | ± | 0.7 |  | 15 | 1.7 | ± | 0.9 |
|  |  |  | Change from Day 0 |  | 19 | -0.1 | ± | 0.5 |  | 15 | 0.5 | ± | 0.9 |
|  |  |  | Group difference [95% CI], *t* test p value |  | 0.5 [0.0, 1.0], p = 0.045^*^ | | | | | | | | |
|  |  |  | ANCOVA group difference estimate [95% CI], p value |  | 0.5 [-0.1, 1.1], p = 0.084 | | | | | | | | |
|  |  | Day 28 | (Score) |  | 18 | 1.3 | ± | 0.7 |  | 16 | 1.7 | ± | 0.9 |
|  |  |  | Change from Day 0 |  | 18 | 0.0 | ± | 0.3 |  | 16 | 0.4 | ± | 0.9 |
|  |  |  | Group difference [95% CI], *t* test p value |  | 0.4 [0.0, 0.9], p = 0.063 | | | | | | | | |
|  |  |  | ANCOVA group difference estimate [95% CI], p value |  | 0.6 [0.1, 1.1], p = 0.023^*^ | | | | | | | | |
| (4)  Conflicting instructions |  | Day 0 | (Score) |  | 19 | 2.4 | ± | 1.1 |  | 16 | 2.3 | ± | 1.1 |
|  |  | Day 14 | (Score) |  | 19 | 2.6 | ± | 0.9 |  | 15 | 2.5 | ± | 1.1 |
|  |  |  | Change from Day 0 |  | 19 | 0.3 | ± | 0.9 |  | 15 | 0.2 | ± | 1.1 |
|  |  |  | Group difference [95% CI], *t* test p value |  | -0.1 [-0.7, 0.6], p = 0.852 | | | | | | | | |
|  |  |  | ANCOVA group difference estimate [95% CI], p value |  | 0.3 [-0.3, 0.8], p = 0.336 | | | | | | | | |
|  |  | Day 28 | (Score) |  | 18 | 2.7 | ± | 0.8 |  | 16 | 2.6 | ± | 0.9 |
|  |  |  | Change from Day 0 |  | 18 | 0.2 | ± | 0.5 |  | 16 | 0.3 | ± | 0.8 |
|  |  |  | Group difference [95% CI], *t* test p value |  | 0.1 [-0.4, 0.5], p = 0.711 | | | | | | | | |
|  |  |  | ANCOVA group difference estimate [95% CI], p value |  | 0.2 [-0.2, 0.6], p = 0.322 | | | | | | | | |
| (5)  Go-no go |  | Day 0 | (Score) |  | 19 | 1.4 | ± | 0.9 |  | 16 | 1.6 | ± | 1.0 |
|  |  | Day 14 | (Score) |  | 19 | 1.7 | ± | 0.9 |  | 15 | 1.6 | ± | 1.1 |
|  |  |  | Change from Day 0 |  | 19 | 0.4 | ± | 1.1 |  | 15 | -0.1 | ± | 0.6 |
|  |  |  | Group difference [95% CI], *t* test p value |  | -0.4 [-1.1, 0.2], p = 0.167 | | | | | | | | |
|  |  |  | ANCOVA group difference estimate [95% CI], p value |  | -0.2 [-0.9, 0.5], p = 0.526 | | | | | | | | |
|  |  | Day 28 | (Score) |  | 18 | 1.6 | ± | 1.0 |  | 16 | 2.1 | ± | 1.0 |
|  |  |  | Change from Day 0 |  | 18 | 0.2 | ± | 1.0 |  | 16 | 0.4 | ± | 0.9 |
|  |  |  | Group difference [95% CI], *t* test p value |  | 0.2 [-0.5, -0.5], p = 0.515 | | | | | | | | |
|  |  |  | ANCOVA group difference estimate [95% CI], p value |  | 0.6 [0.0, -1.3], p = 0.061 | | | | | | | | |
| (6)  Prehension behavior |  | Day 0 | (Score) |  | 19 | 3.0 | ± | 0.0 |  | 16 | 3.0 | ± | 0.0 |
|  |  | Day 14 | (Score) |  | 19 | 3.0 | ± | 0.0 |  | 15 | 3.0 | ± | 0.0 |
|  |  |  | Change from Day 0 |  | 19 | 0.0 | ± | 0.0 |  | 15 | 0.0 | ± | 0.0 |
|  |  |  | Group difference [95% CI], *t* test p value |  | --- | | | | | | | | |
|  |  |  | ANCOVA group difference estimate [95% CI], p value |  | --- | | | | | | | | |
|  |  | Day 28 | (Score) |  | 18 | 3.1 | ± | 0.2 |  | 16 | 3.0 | ± | 0.0 |
|  |  |  | Change from Day 0 |  | 18 | 0.1 | ± | 0.2 |  | 16 | 0.0 | ± | 0.0 |
|  |  |  | Group difference [95% CI], *t* test p value |  | -0.1 [-0.2, 0.1], p = 0.354 | | | | | | | | |
|  |  |  | ANCOVA group difference estimate [95% CI], p value |  | -0.1 [-0.2, 0.1], p = 0.205 ^#^ | | | | | | | | |

Group difference: differences between groups (amino acid mixture group - placebo group) in the change from Day 0 and their 95% confidence intervals (95% CI). t test: comparison between groups in the change from Day 0 by unpaired *t* test. ANCOVA: analysis of covariance adjusted for baseline value (Day 0), years of education and ApoE4 type. *: P<0.05. #: ANCOVA results excluding baseline values from the covariates because the baseline values of all subjects were the same. SD: standard deviation, FAB: Frontal Assessment Battery. For the results displayed as "---", there was no variation in the data, and statistical tests could not be performed.
